# *In vivo* evaluation of cellular activity in *α*CaMKII heterozygous knockout mice using manganese-enhanced magnetic resonance imaging (MEMRI)

**DOI:** 10.3389/fnint.2013.00076

**Published:** 2013-11-11

**Authors:** Satoko Hattori, Hideo Hagihara, Koji Ohira, Ichio Aoki, Tsuneo Saga, Tetsuya Suhara, Makoto Higuchi, Tsuyoshi Miyakawa

**Affiliations:** ^1^Molecular Neuroimaging Program, Molecular Imaging Center, National Institute of Radiological SciencesChiba, Japan; ^2^Division of Systems Medical Science, Institute for Comprehensive Medical Science, Fujita Health UniversityToyoake, Aichi, Japan; ^3^Japan Science and Technology Agency (JST), Core Research for Evolutional Science and Technology (CREST)Kawaguchi, Saitama, Japan; ^4^Diagnostic Imaging Program, Molecular Imaging Center, National Institute of Radiological SciencesChiba, Japan; ^5^Center for Genetic Analysis of Behavior, National Institute for Physiological SciencesOkazaki, Aichi, Japan

**Keywords:** *α*CaMKII, manganese-enhanced MRI, immature, dentate gyrus, hippocampus, bed nucleus of stria terminalis, schizophrenia, psychiatric disorder

## Abstract

The alpha-calcium/calmodulin-dependent protein kinase II (αCaMKII) is a serine/threonine protein kinase predominantly expressed in the forebrain, especially in the postsynaptic density, and plays a key role in synaptic plasticity, learning and memory. αCaMKII heterozygous knockout (HKO) mice exhibit abnormal emotional and aggressive behaviors and cognitive impairments and have been proposed as an animal model of psychiatric illness. Our previous studies have shown that the expression of immediate early genes (IEGs) after exposure to electric foot shock or after performing a working memory task is decreased in the hippocampus, central amygdala, and medial prefrontal cortex of mutant mice. These changes could be caused by disturbances in neuronal signal transduction; however, it is still unclear whether neuronal activity is reduced in these regions. In this study, we performed *in vivo* manganese-enhanced magnetic resonance imaging (MEMRI) to assess the regional cellular activity in the brains of αCaMKII HKO mice. The signal intensity of MEMRI 24 h after systemic MnCl_2_ administration reflects functional increases of Mn^2+^ influx into neurons and glia via transport mechanisms, such as voltage-gated and/or ligand-gated Ca^2+^ channels. αCaMKII HKO mice demonstrated a low signal intensity of MEMRI in the dentate gyrus (DG), in which almost all neurons were at immature status at the molecular, morphological, and electrophysiological levels. In contrast, analysis of the signal intensity in these mutant mice revealed increased activity in the CA1 area of the hippocampus, a region crucial for cognitive function. The signal intensity was also increased in the bed nucleus of the stria terminalis (BNST), which is involved in anxiety. These changes in the mutant mice may be responsible for the observed dysregulated behaviors, such as cognitive deficit and abnormal anxiety-like behavior, which are similar to symptoms seen in human psychiatric disorders.

## Introduction

The alpha isoform of calcium/calmodulin-dependent protein kinase II (*α*CaMKII) is a calcium-activated, serine/threonine protein kinase and is abundant in the brain. It is enriched at the postsynaptic density (Lisman et al., 2002), and its activity is necessary for long-term potentiation of synaptic transmission in the hippocampus that may regulate learning and memory. Previous studies have shown that spatial learning and memory are affected in both homozygous and heterozygous *α*CaMKII knockout mice (Silva et al., 1992, 1996; Gordon et al., 1996; Frankland et al., 2001; Elgersma et al., 2002), as well as in several strains of *α*CaMKII transgenic mice (reviewed in Elgersma et al., 2004).

*α*CaMKII heterozygous knockout (HKO) mice also have various behavioral abnormalities that resemble symptoms seen in human psychiatric disorders, including decreased fear response, enhanced defensive aggression (Chen et al., 1994), increased locomotor activity, deficit in working memory, high level of social aggression toward cage mates, and an exaggerated infradian rhythm (Yamasaki et al., 2008). We found that molecular, morphological, and electrophysiological features in the dentate gyrus (DG) neurons of adult mutant mice were similar to those of immature DG neurons in normal rodents (Yamasaki et al., 2008). The “immature dentate gyrus (iDG)” phenotype has been observed in the post-mortem brains of patients with schizophrenia and bipolar disorder (Walton et al., 2012), as well as in other mouse models of these disorders (Hagihara et al., 2011; Ohira et al., 2013; Takao et al., 2013). In addition, levels of dopamine D2 receptors in a state with a high affinity for dopamine (D2^High^ receptors) were found to be elevated in the striatum of *α*CaMKII HKO mice, which could be representative of the hyperdopaminergic state seen in patients with schizophrenia (Novak and Seeman, 2010). Therefore, it has been proposed that *α*CaMKII HKO mice are promising animal models of schizophrenia and other psychiatric disorders (Yamasaki et al., 2008; Novak and Seeman, 2010) and that the iDG might serve as a novel endophenotype of the disorders, (Walton et al., 2012; Hagihara et al., 2013).

Our previous studies have shown that *α*CaMKII HKO mice had marked abnormalities in neurotransmitter binding to their receptors and neuronal activity in several brain regions (Yamasaki et al., 2008; Matsuo et al., 2009). Quantification of the expression of immediate-early genes (IEGs), which are activated in response to neuronal stimuli, exhibited lower expression levels of c-Fos in the DG, CA3, and central amygdaloid nucleus of the mutant mice than those of the wild-type mice following electric foot shock (Yamasaki et al., 2008). In the *α*CaMKII HKO mice, following a working memory version of the eight-arm radial maze task, the expressions of c-Fos were reduced in neurons of the hippocampal DG, CA1, and CA3 areas, central amygdala, and medial prefrontal cortex, whereas their expressions in the mutant mice kept in home cages were decreased in the DG, but not in other areas (Matsuo et al., 2009). In addition, the neurons in the mutant DG had abnormal electrophysiological features, including high excitability, small spike amplitude, and a decreased number of spikes during sustained depolarization (Yamasaki et al., 2008). These results suggest that *α*CaMKII HKO mice have functional deficits in several brain regions. However, it is possible that the altered expression of such IEGs is caused by a disruption in the signaling pathways that link neuronal activity to transcription, and it remains unclear whether neuronal activity is also altered in these regions of the mutant mice.

In this study, we evaluated the brains of *α*CaMKII HKO mice using systemically Mn^2+^ administrated MEMRI without blood-brain-barrier disruption (Watanabe et al., 2001; Aoki et al., 2004). This is an effective method to detect and visualize the anatomical and functional features of the brain. Mn^2+^ is a positive contrast agent for MRI and can accumulate in excitable cells via some of the transport mechanisms shared with calcium, such as voltage-gated Ca^2+^ channels and ionotropic glutamate receptors (Itoh et al., 2008; Silva and Bock, 2008; Hankir et al., 2012). The signal intensity on a T_1_-weighted (T_1_W) MR image is enhanced by Mn^2+^ uptake through activated ion channels; therefore, this technique can reflect the cellular activity in brain regions (Yu et al., 2005). We performed MRI scans on *α*CaMKII HKO and wild-type mice 1 day after systemic MnCl_2_ intravenous administration, and assessed the normalized signal intensity under baseline conditions in the home cage.

## Materials and methods

### Animals and experimental design

*α*CaMKII HKO mice generated by gene-targeting techniques were obtained from Jackson Laboratories (Bar Harbor, ME, USA). Mice were housed one per cage in a room with a 12 h light dark cycle (light on at 7:00 a.m.) with access to food and water *ad libitum*. MEMRI was performed on 7 to 10 month-old *α*CaMKII HKO mice (*n* = 7) and wild-type littermates (*n* = 7) on a C57BL/6J background. The Institutional Animal Care and Use Committee of the National Institute of Radiological Sciences and Fujita Health University approved the present experimental protocol.

### Manganese administration

Prior to the administration, 100 mM of MnCl_2_ (MnCl_2_-4H_2_O, Sigma-Aldrich, St. Louis, MO, USA) was made with distilled water and diluted to 50 mM with saline to match the osmotic pressure of blood. We slowly infused 75 mg/kg (380 μmol/kg) MnCl_2_ (total volume: 0.2–0.3 mL) for 60 min through the tail vein using a syringe pump (KDS-100, KD Scientific, Holliston, MA, USA). The MnCl_2_ dose used in this study provided clear regional contrast and was similar to the doses used in previous MEMRI studies of the mouse brain (Yu et al., 2005; Lutkenhoff et al., 2012; Perez et al., 2013). After MnCl_2_ injection, each mouse showed reduced locomotor activity temporarily for approximately 2–3 h, probably due to the toxic effect of MnCl_2_. We did not notice any apparent differences in the behavioral response to MnCl_2_ between genotypes. Mice were kept anesthetized with 0.5–1.5% isoflurane (Mylan Inc., Tokyo, Japan) during MnCl_2_ infusion. Rectal temperature was continuously monitored and automatically maintained at approximately 37.5°C using a temperature controller (E5GN, Omron, Inc., Kyoto, Japan) and electrical heating pad (SG-15, Showa-Seiki industry, Inc., Kobe, Japan).

### Animal preparation and MRI measurements

We performed MRI in a 7.0 Tesla scanner, with a 40 cm bore magnet (Kobelco and Jastec, Tokyo, Japan) interfaced with a Bruker Avance-I console (BioSpec, Bruker Biospin, Ettlingen, Germany) with a volume coil for transmission (Bruker Biospin, Ettlingen, Germany) and a two-channel phased-array coil for reception (Rapid Biomedical, Rympar, Germany). Mice were anesthetized with 1.0–2.0% isoflurane and placed in prone position. During the experiment, a warm airflow over the animal was used to maintain its rectal temperature at 37.5°C. Respiratory rate was maintained at 20–40 breaths per minute and monitored throughout the experiment. Two-dimensional single-slice T_1_W images were obtained by conventional spin-echo sequence with the following parameters: pulse repetition time (TR) = 250 ms; echo time (TE) = 9.574 ms; matrix size = 192 × 192; field of view = 1.92 × 1.92 cm^2^; slice thickness = 1.0 mm; slice gap = 1 mm; spatial resolution = 100 × 100 × 1000 μm^3^; and number of acquisitions = 4. A complete set of T_1_W measurements consisted of two T_1_W scans with slice offsets of 0 and 1 mm to maintain continuity of slices and to cover the entire brain. To register the image plane exactly, anatomical scout images were acquired using an incoherent, gradient-echo, fast low-angle shot sequence (TR = 100 ms; TE = 6 ms; matrix = 256 × 256; slice thickness = 2.0 mm). The slice orientation of the coronal plane was carefully adjusted on the sagittal scout image according to the landmarks of the pituitary body with reference to a mouse brain atlas (Paxinos and Franklin, 2001).

### MRI data analysis

MR image data were converted from native Bruker format to voistat and TIFF files using PMOD (version 2.6; PMOD Technologies, Zurich, Switzerland), and analyzed quantitatively with PMOD and ImageJ.^1^ Through comparison of MR images with a mouse brain atlas (Paxinos and Franklin, 2001), regions of interest (ROIs) for quantitative analysis of MEMRI were defined and delineated manually in the hippocampus, bed nucleus of the stria terminalis (BNST), cortex, striatum, thalamus, midbrain, and amygdala. We compared the MR image with the atlas based on the distance from the pituitary. Signal intensity was measured in each ROI, and we present the data normalized to that in the whole brain (Perez et al., 2013). In many cases, a slight signal intensity gradient was observed that could change within a plane and serve as noise. To minimize such noise, we used the average signal intensity of whole brain for normalization.

Statistical analyses were conducted using StatView software (SAS Institute, Cary, NC, USA). All data are presented as the mean ± the standard error of the mean (SEM), and were analyzed by one-way analysis of variance (ANOVA). An alpha level adjusted for multiple comparisons was calculated for each brain region by Bonferroni-Holm method.

### Quantification of cell number

Adult *α*CaMKII HKO mice (*n* = 3) and wild-type littermates (*n* = 3) were used. They were perfused through the heart with ice-cold phosphate buffered saline (PBS) and then with 4% paraformaldehyde (PFA) in 0.1 M PBS, with pH 7.4. After perfusion, the brains were immediately removed and immersed in the same fixative at 4°C overnight, followed by successive immersions in 30% sucrose in PBS. The brains were mounted in Tissue-Tek (Miles Inc., Elkhart, NY, USA), frozen, and stored at −80°C until use. Brains were sliced coronally into 10 μm thick sections on a cryostat (CM1850, Leica Microsystems, Wetzlar, Germany). The sections were stained with Hoechst 33258 (Polyscience, Warrington, PA, USA). Fluorescent signals were detected using a confocal laser-scanning microscope (LSM 700, Zeiss, Oberkochen, Germany). For the quantification of region size and Hoechst-stained cell numbers, we used ImageJ with the WCIF ImageJ bundle^2^ (Takao et al., 2013). Three sections obtained from the anterior hippocampal region (from bregma −1.70 mm to bregma −2.30 mm, approximately) per animal were examined. ROI were delineated manually on the Hoechst-stained images with reference to the mouse brain atlas (Paxinos and Franklin, 2001). The values were then averaged within each brain and by group. All data collected in quantitative analyses were statistically evaluated using Student’s *t*-test for comparison of means.

## Results

**Figure 1:**
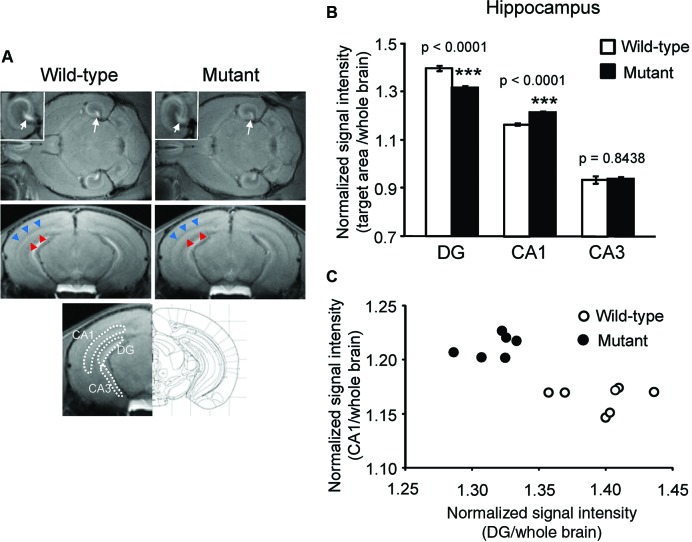
**Signal intensity decreased in the DG and increased in the CA1 region of *α*CaMKII HKO mice**. **(A)** Representative T_1_W MR images after systemic MnCl_2_ administration. The top row shows horizontal slices at the level of the hippocampus. White squares indicate areas of the hippocampus presented at a high magnification. White arrows indicate the V-shaped structure of the DG. The middle row shows coronal slices including the hippocampal structure (bregma: −3.08 mm). The CA1 and stratum radiatum, including the projection area of the CA3, are indicated by red and blue arrowheads, respectively. The bottom row shows the anatomical location of ROIs in the hippocampus. The ROIs correspond to the DG, CA1, and CA3, respectively. **(B)** Normalized signal intensities in the hippocampal subregions are presented as the mean ± SEM. The *p* values indicate genotype effect in one-way ANOVA. **(C)** Scatterplot of normalized signal intensity in the DG versus CA1 region.

Normalized signal intensities of MEMRI responses in several anatomically defined ROIs were calculated semiquantitatively in *α*CaMKII HKO and wild-type mice. Figure 1A shows T_1_W MR images of horizontal and coronal slices 1 day after MnCl_2_ administration. The normalized signal intensity in MEMR images was significantly lower in the DG of mutant mice than in the DG of wild-type mice (Figures 1A, B, mutant vs. wild-type: 1.317 ± 0.007 vs. 1.397 ± 0.012, *F*_1,12_ = 46.994, *p* < 0.0001; adjusted *α* = 0.0063). In contrast, the CA1 field including stratum radiatum, which is a projection area of the CA3, showed higher signal enhancement in *α*CaMKII HKO mice (Figure 1A). Normalized signal intensity was significantly higher in the CA1 of mutant mice than in the CA1 of wild-type mice (Figure 1B, mutant vs. wild-type: 1.215 ± 0.005 vs. 1.165 ± 0.005, *F*_1,12_ = 71.086, *p* < 0.0001; adjusted *α* = 0.0056). However, there was no significant difference in normalized signal intensity in the CA3 region between the two genotypes (Figure 1B, mutant vs. wild-type: 0.937 ± 0.008 vs. 0.934 ± 0.016, *F*_1,12_ = 0.041, *p* = 0.8438). A scatter plot showing the relationship between normalized signal intensity in the DG and CA1 field (Figure 1C) indicates that the subregional profile of hippocampal cellular activity in the *α*CaMKII HKO mice is clearly distinct from that in the normal mice.

Signal intensity in MEMRI could reflect cell density in addition to cellular activity. The cell density in the mutant mice was assessed by Hoechst stain. In the DG, a significant increase in cell density was detected in the mutant mice (mutant vs. wild-type: 2357 ± 83 vs. 1578 ± 57, *p* = 0.0012) as compared to in the wild-type mice, which is likely the result of greatly increased adult neurogenesis in the DG of mutant mice (Yamasaki et al., 2008). There were no significant differences in cell density in the CA1 (mutant vs. wild-type: 1256 ± 8 vs. 1234 ± 45, *p* = 0.2026) and CA3 fields (mutant vs. wild-type: 552 ± 6 vs. 581 ± 18, *p* = 0.4926) between genotypes. In general, signal intensity in MEMRI is expected to be positively correlated with cell density (Silva et al., 2004), given that there is no difference in cellular activity. However, in the mutant mice, the signal intensity of the DG decreased, while cell density increased. These results suggest that decreased signal intensities in mutant mice are due to decreased cellular activity, not to increased cell density. Total cell numbers counted were 112.3 ± 11.9 in wild-type mice and 159.3 ± 2.9 in mutant mice in the dorsal part of the granule cell layer (*p* = 0.0183), 87.7 ± 7.5 in wild-type mice and 88.7 ± 3.2 in mutant mice in the pyramidal cell layer of CA1 (*p* = 0.9084), and 130.7 ± 7.5 in wild-type mice and 154.7 ± 6.6 in mutant mice in the pyramidal cell layer of CA3 (*p* = 0.0742).

**Figure 2:**
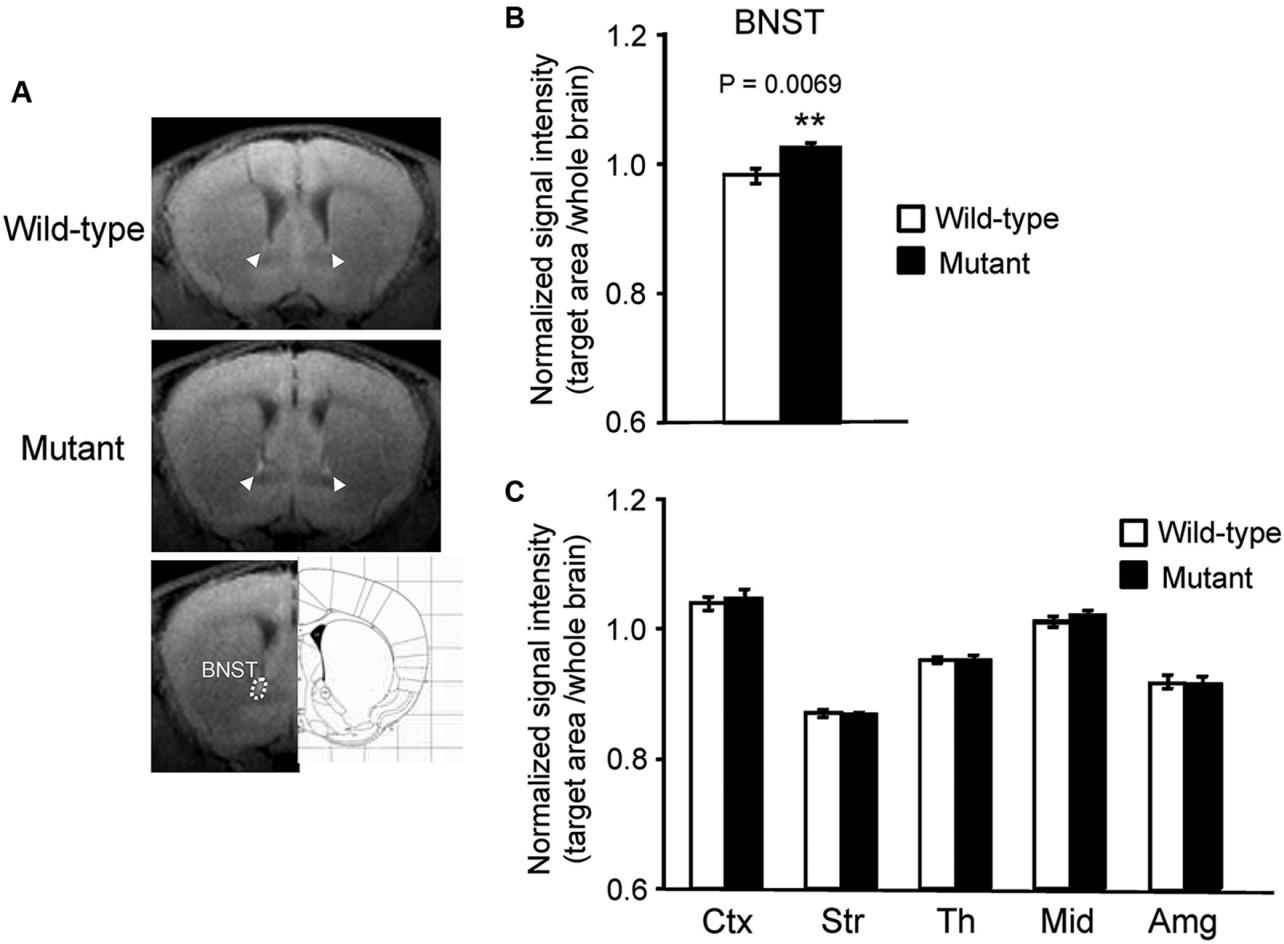
**Signal intensity increased in the BNST of *α*CaMKII HKO mice**. **(A)** Representative T_1_W MR images after systemic MnCl_2_ administration. The top and middle rows show coronal slices at the level of the BNST (bregma: + 0.62 mm). The BNST is indicated by white arrowheads. The bottom row shows the anatomical location of ROIs in the BNST. **(B)** Normalized signal intensity in the BNST is presented as the mean ± SEM for the indicated numbers of animals **(C)** Normalized signal intensities in other regions of the brain are presented as the mean ± SEM. The *p* values indicate the effect of genotype in one-way ANOVA.

In addition to the hippocampus, we observed increased MEMRI signal in the BNST of *α*CaMKII HKO mice (Figure 2A), and normalized signal intensity of the BNST was significantly higher in mutant mice than in wild-type mice (Figure 2B, mutant vs. wild-type: 1.024 ± 0.009 vs. 0.983 ± 0.012, *p* = 0.0069; adjusted *α* = 0.0071). We also estimated normalized signal intensity in the major regions of the brain, such as the cortex, striatum, thalamus, midbrain, and amygdala. No significant differences were observed in the signal intensity of MEMRI in these regions between genotypes (Figure 2C, cortex: *F*_1,12_ = 0.174, *p* = 0.6836; striatum: *F*_1,12_ = 0.090, *p* = 0.7691; thalamus: *F*_1,12_ = 0.005, *p* = 0.9442; midbrain: *F*_1,12_ = 1.390, *p* = 0.2613; amygdala: *F*_1,12_ = 0.127, *p* = 0.7273). We also analyzed the data using signal intensities of the cortex or striatum for normalization, which yielded essentially the same results as those derived from the analysis using the signal intensities of the whole brain for normalization (data not shown).

## Discussion

The *α*CaMKII HKO mouse has been proposed as an animal model of psychiatric illnesses, including schizophrenia and bipolar disorders. In this study, MEMRI demonstrated that Mn^2+^ accumulation was reduced in the DG and elevated in the BNST and CA1 field of the hippocampus.

We previously reported that *α*CaMKII HKO mice exhibited the iDG phenotype, a potential brain endophenotype shared by patients with schizophrenia and bipolar disorder (Walton et al., 2012; Hagihara et al., 2013; Shin et al., 2013). In the mutant DG, IEGs expression is abolished almost completely following a working memory task and electric foot shocks (Yamasaki et al., 2008; Matsuo et al., 2009), and electrophysiological evidence has revealed a decrease in the number of spikes during sustained depolarization (Yamasaki et al., 2008). These findings suggest that there is a decrease in neuronal activity and/or a disruption in the signal transduction pathway required to induce IEGs in the mutant DG. In this study, we examined the activity profile in the brains of mutant mice by performing MEMRI. A lower signal intensity of MEMRI was observed in the V-shaped structure of the DG in *α*CaMKII HKO mice than in that of the wild-type mice, and this might be the result of reduced activity in DG granule cells. Several lines of evidence support the reduction of activity in the DG. Downregulation in the expression levels of Ca^2+^-permeable receptors could lead to the low activity observed in the DG of mutant mice. We have reported a decrease in N-methyl-D-aspartate (NMDA) receptor binding by using autoradiographic techniques (Yamasaki et al., 2008). Decreased Mn^2+^ influx through these receptors could be detected on the MEMRI as differences in Mn^2+^-enhanced signal intensity in the granule cells of mutant mice. Alternatively, the activation of local circuits by immature neurons in the DG could inhibit the activity of the entire DG. Lacefield et al. proposed the possibility that immature granule cells effectively drive hilar interneurons, which innervate back to all granule cells (Lacefield et al., 2012). Thus, an excess of immature neurons may cause gross inhibition of the DG by a feedback loop and result in the reduced enhancement of MRI signal intensity observed in the DG of mutant mice.

In contrast to the DG, the CA1 field of the *α*CaMKII HKO mice showed greater Mn^2+^ uptake, especially the stratum radiatum, which contains projection fibers to CA1 neurons. This result supports the idea of increased activity in the CA1 field of mutant mice. One possibility is that the augmentation in CA1 neuronal activity could be caused by an altered response to inputs from the entorhinal cortex (EC). The hippocampus mainly includes two excitatory networks, the monosynaptic pathway (from layer III in EC to CA1) and the trisynaptic pathway (from layer II in EC to DG to CA3 to CA1) (Amaral and Witter, 1989). Dysfunction in the mutant DG could reduce neuronal transmission in the trisynaptic pathway, and, therefore, homeostatic regulation of hippocampal network activity might lead to an increase in monosynaptic excitation of the CA1 region. Additionally, the decreased activity in the DG of these mice might cause activation in the CA3 and then in the CA1 field. Mossy fibers from the DG release the excitatory neurotransmitter glutamate (Henze et al., 2000). In the CA3, mossy fibers project to GABAergic interneurons that provide inhibitory inputs to CA3 pyramidal cells, and also terminate in CA3 pyramidal neurons (Henze et al., 2000). Activity of the DG and mossy fibers may result in a net inhibition within the CA3 network and presumably activate only a specific subset of CA3 pyramidal neurons (Henze et al., 2000; Song et al., 2012). In the *α*CaMKII HKO mice, decreased DG activity may lead to a tonic net activation of the CA3 and CA1 through a trisynaptic pathway. In this study, no difference in the MEMRI signal intensity ratio between genotypes could be detected in the CA3 area. This might result from limitations related to our methodology, including spatial resolution and detection sensitivity. Finally, the increased activity might be an effect of the HKO of *α*CaMKII itself in cells present in the CA1 area. Our previous study demonstrated a downregulation in serotonin 1A receptor binding and an upregulation of serotonin transporter binding in the CA1 of mutant mice (Yamasaki et al., 2008). Such abnormalities in the CA1 may have caused the increased activity. Further detailed studies, combined with *in vivo* imaging and electrophysiological methods, are needed to address the activity in each hippocampal subregion of the mutant mice.

A previous study revealed that the area showing enhanced signal on Mn^2+^-contrasted MRI corresponds to the area that exhibits increased expression of IEGs (Morita et al., 2002). In the DG, our result is consistent with this observation. However, there is a discrepancy between MEMRI signal intensity and c-Fos expression in the CA1. The expression of c-Fos following a working memory task was decreased in the mutant (Matsuo et al., 2009), and there was no significant difference in its expression in the CA1 of both genotypes after electrical foot shock and when the mice were kept in the home cage (Yamasaki et al., 2008; Matsuo et al., 2009). The inconsistency may be due to signal transduction abnormalities in IEG expression and/or to differences in experimental conditions, including the type of stimuli presented to the mice.

Additionally, previous studies suggested that the accumulation of reactive astrocytes and/or the migration of microglia could also cause increased signal intensity of MEMRI (Widerøe et al., 2009; Kawai et al., 2010). In *α*CaMKII HKO mice, the expression of glial fibrillary acidic protein, a well-established marker of astrocytes, is increased in the DG and CA1 area, especially the stratum radiatum and stratum oriens (unpublished data). In contrast, no significant difference between genotypes was observed in the immunoreactivity of ionized calcium binding adaptor molecule 1, a microglia/macrophage-specific calcium binding protein, in either the DG or CA1 field (unpublished data). These data suggest that decreased Mn^2+^ uptake in the mutant DG could be mainly due to altered neuronal activity. On the other hand, it is possible that the increased signal intensity of MEMRI, which was observed in the CA1 of mutant mice, was influenced by an increase in reactive astrocytes as well as by changes in neuronal activity.

In *α*CaMKII HKO mice, altered signal intensity in MEMRI was observed in the hippocampus, a region that plays a key role in cognitive functions such as working memory (Goldman-Rakic, 1994) and pattern separation (Gilbert et al., 2001; Gilbert and Kesner, 2003; Lee and Kesner, 2004a,b; Leutgeb et al., 2007; McHugh et al., 2007; Nakashiba et al., 2008). Impairments in hippocampal function are often observed in patients with psychiatric disorders such as schizophrenia and anxiety disorders (Goldman-Rakic, 1994) and may represent a behavioral endophenotype of these disorders. We demonstrated that *α*CaMKII HKO mice had severe deficits in working memory (Yamasaki et al., 2008), and that the density of c-Fos-positive cells in the hippocampus after a working memory task was significantly lower in the mutant mice than in the wild-type mice (Matsuo et al., 2009). In the EC of mutant mice, IEG expression was induced to the same extent as that observed in wild-type mice after the task. Thus, reduced or aberrant neuronal activity in the DG, which is a key input node for the hippocampal trisynaptic pathway, might lead to altered hippocampal network activity and impaired working memory performance in mutant mice. Indeed, previous studies have shown that rats with lesions of the DG are impaired in their ability to perform a working memory version of the eight-arm radial maze task (McLamb et al., 1988; Emerich and Walsh, 1989; Morris et al., 2012). It is possible that working memory dysfunction in mutant mice is caused by impaired rapid encoding of spatial information.

The mutant mice also showed increased MEMRI signal intensity in the BNST. This region has roles in the regulation of the hypothalamic-pituitary-adrenal axis response to acute stress (Choi et al., 2007). Recent studies in mice demonstrated that the oval nucleus increases anxiety-like behavior, while anterodorsal subregions of the BNST decrease anxiety-like behavior (Kim et al., 2013), and that BNST excitatory and inhibitory projections differentially produce anxiogenic and anxiolytic behavioral phenotypes, respectively (Jennings et al., 2013). These findings suggest that BNST acts as a crucial circuit node for bidirectional regulation of anxiety related responses. It is possible that increased BNST activity underlies decreased anxiety-like behavior in the *α*CaMKII mutant mice. During a casual visual inspection of the images, we noticed that the signal intensity was obviously increased in a discrete region of the BNST. There is a possibility that we could detect differences in signal intensities in other brain subregions as well. Future studies, using voxel-based analyses, are needed to assess the differences in detail.

In this study, we evaluated *in vivo* cellular activity in the brains of *α*CaMKII HKO mice by using MEMRI. It has been proposed that these mice are useful animal models of neuropsychiatric disorders (Yamasaki et al., 2008; Novak and Seeman, 2010; Hagihara et al., 2013; Shin et al., 2013). Our study is the first application of *in vivo* MEMRI in the mutant mice that focused on baseline activity rather than stimulus driven activity. Reduced excitatory signaling in the DG and increased activity in the CA1 appear to be associated with the pathological features of schizophrenia (Schobel et al., 2009; Tamminga et al., 2010). The results of the current study also suggest that cellular activity decreases in the DG and increases in the CA1 of mutant mice, providing additional support for the validity of these mice as an animal model of these disorders. The iDG and abnormal behavioral phenotypes are shared with Shn-2 knockout (Takao et al., 2013), mutant SNAP-25 knock-in (Ohira et al., 2013), and forebrain-specific calcineurin knockout mice (unpublished data). Chronic fluoxetine treatment and pilocarpine-induced seizures also lead to the iDG phenotype (Kobayashi et al., 2010; Shin et al., 2013). It is of interest to examine whether these mice show similar patterns of Mn^2+^ accumulation in MEMRI. Moreover, recent studies have demonstrated regional alteration of cellular activity in other animal models of neuropsychiatric disorders (Lutkenhoff et al., 2012; Perez et al., 2013). In addition to these studies, our results suggest that non-invasive MRI measurement is applicable for translational research of neuropsychiatric disorders. MEMRI of mutant mice would also provide biological outcome measures in screening of novel therapeutic compounds targeting these disorders.

## Conflict of interest statement

Tsuyoshi Miyakawa is an advisor/consultant for Astellas Pharma Inc. The other authors declare that the research was conducted in the absence of any commercial or financial relationships that could be construed as a potential conflict of interest.

## Footnotes

^1^http://rsb.info.nih.gov/ij/

^2^http://www.uhnres.utoronto.ca/facilities/wcif/
